# Supplementary material for: Rare variant associations with birth weight identify genes involved in adipose tissue regulation, placental function and insulin-like growth factor signalling
Source: Nat Commun. 2025 Jan 14;16:648. doi: 10.1038/s41467-024-55761-2 (PMC11733218; doi:10.1038/s41467-024-55761-2)
Supplement: Supplementary file 2 — Description of Additional Supplementary Files [file 41467_2024_55761_MOESM2_ESM.pdf]

Description of Additional Supplementary Files

File Name: Supplementary Data 1

Description: Main exome assocs

We tested genes with at least 10 carriers of rare (frequency <0.1%) variants that were either either high confidence protein truncating variants (HC PTV) or damaging variants, defined as either HC PTVs or missense variants with a CADD score >=25. Genes passing the exome-wide significance and multiple test-corrected threshold of $P<1.64\times10\text{-}6$ in the fetal analysis and $P<1.58\times10\text{-}6$ in the maternal analysis (two-sided), in the Discovery BOLT-LMM analyses, were then independently confirmed using REGENIE.

File Name: Supplementary Data 2

Description: WLMs, maternal vs fetal

A weighted linear model (WLM) was applied for each of the exome-wide significantly associated genes and their associations in the fetal and maternal analyses (two-sided comparison).

File Name: Supplementary Data 3

Description: Sexual dimorphism

Sex-specific effects (two-sided comparison) of the genes identified in the fetal analysis.

File Name: Supplementary Data 4

Description: deCODE replication

Replication of all exome-wide significantly associated genes in an independent cohort of Icelandic data (two-sided test).

File Name: Supplementary Data 5

Description: GWAS signals

Lookup of all exome-wide significantly associated genes for proximal GWAS signals, at a multiple-test corrected threshold of $P<5x10\text{-}8$ in the birthweight GWASs from Juliusdottir et al. (two-sided test) with accompanying eQTL information from GTEx.

File Name: Supplementary Data 6

Description: GWAS signal conditional

For genes proximal to GWAS signals, the associations from ST1 were conditioned on the genotypes of the GWAS signals from ST5, in a linear model (two-sided) framework.

File Name: Supplementary Data 7

Description: Exome assocs at GWAS

Table of genes within 300kb of a GWAS signal passing a Benjamini-Hochberg P-value correction of 0.05. P_adj is the P value adjusted for multiple testing by a Benjamini-Hochberg FDR criteria.

File Name: Supplementary Data 8

Description: GWAS pathway enrichment

Pathway enrichment analyses for the maternal and fetal GWAS genes from GWASs from Juliusdottir et al. using gprofiler.

File Name: Supplementary Data 9

Description: PheWAS

Small-scale PheWAS of all exome-wide significantly associated genes, in related metabolic phenotypes, from either BOLT-LMM or REGENIE (two-sided tests).

File Name: Supplementary Data 10

Description: INHBE

Leave-one-out sensitivity analyses for the splice variant in INHBE, in a linear model (two-sided) framework.

File Name: Supplementary Data 11

Description: PPARG

Bioinformatically and experimentally selected missense variants in PPARG were concomittantly tested for associations in linear models (two-sided) towards birthweight (fetal analysis) and adult body fat percentage.

File Name: Supplementary Data 12

Description: GCK

Known pathogenic variants in GCK were compared to annotated PTVs and missense variants, for their maternal and fetal effect on birthweight, using REGENIE (two-sided).
