# Supplementary material for: Rare variant associations with birth weight identify genes involved in adipose tissue regulation, placental function and insulin-like growth factor signalling
Source: Nat Commun. 2025 Jan 14;16:648. doi: 10.1038/s41467-024-55761-2 (PMC11733218; doi:10.1038/s41467-024-55761-2)
Supplement: Supplementary file 4 — Reporting Summary [file 41467_2024_55761_MOESM4_ESM.pdf]

nature portfolio

Corresponding author(s): Rachel Freathy

Last updated by author(s): Nov 21, 2024

Reporting Summary

Nature Portfolio wishes to improve the reproducibility of the work that we publish. This form provides structure for consistency and transparency in reporting. For further information on Nature Portfolio policies, see our Editorial Policies and the Editorial Policy Checklist.

Statistics

For all statistical analyses, confirm that the following items are present in the figure legend, table legend, main text, or Methods section.

n/a	Confirmed	
☐	☒	The exact sample size (*n*) for each experimental group/condition, given as a discrete number and unit of measurement
☒	☐	A statement on whether measurements were taken from distinct samples or whether the same sample was measured repeatedly
☐	☒	The statistical test(s) used AND whether they are one- or two-sided<br>*Only common tests should be described solely by name; describe more complex techniques in the Methods section.*
☐	☒	A description of all covariates tested
☐	☒	A description of any assumptions or corrections, such as tests of normality and adjustment for multiple comparisons
☐	☒	A full description of the statistical parameters including central tendency (e.g. means) or other basic estimates (e.g. regression coefficient) AND variation (e.g. standard deviation) or associated estimates of uncertainty (e.g. confidence intervals)
☐	☒	For null hypothesis testing, the test statistic (e.g. *F*, *t*, *r*) with confidence intervals, effect sizes, degrees of freedom and *P* value noted<br>*Give P values as exact values whenever suitable.*
☒	☐	For Bayesian analysis, information on the choice of priors and Markov chain Monte Carlo settings
☒	☐	For hierarchical and complex designs, identification of the appropriate level for tests and full reporting of outcomes
☐	☒	Estimates of effect sizes (e.g. Cohen's *d*, Pearson's *r*), indicating how they were calculated

Our web collection on statistics for biologists contains articles on many of the points above.

Software and code

Policy information about availability of computer code

Data collection	No software used for data collection.
Data analysis	ENSEMBL VEP v104, BOLT-LMM v2.3.6, plink v1.90b6.18, R v4.2.1, ggplot2 v3.3.6, CADD v1.6, REGENIE v3.1.3, gprofiler2 v0.2.1, GraphTyper v2.6, SHAPEIT4

For manuscripts utilizing custom algorithms or software that are central to the research but not yet described in published literature, software must be made available to editors and reviewers. We strongly encourage code deposition in a community repository (e.g. GitHub). See the Nature Portfolio guidelines for submitting code & software for further information.

Data

Policy information about availability of data

All manuscripts must include a data availability statement. This statement should provide the following information, where applicable:

- Accession codes, unique identifiers, or web links for publicly available datasets
- A description of any restrictions on data availability
- For clinical datasets or third party data, please ensure that the statement adheres to our policy

Data from the UK Biobank are available by application to all bona fide researchers in the public interest at https://www.ukbiobank.ac.uk/enable-your-research/apply-for-access. Additional information about registration for access to the data is available at www.ukbiobank.ac.uk/register-apply/.

Research involving human participants, their data, or biological material

Policy information about studies with human participants or human data. See also policy information about sex, gender (identity/presentation), and sexual orientation and race, ethnicity and racism.

Reporting on sex and gender	In our analyses we included data from both male and female study participants and we adjusted sex in our analyses.
Reporting on race, ethnicity, or other socially relevant groupings	In UK Biobank, we restricted our analysis to individuals of European ancestry, and we defined a subset of European ancestry samples using a k-means-clustering approach that was applied to the first four principal components calculated from genome-wide SNP genotypes.
Population characteristics	The UK Biobank is a large prospective cohort that recruited approximately 500,000 participants aged 40 to 69 years across the island of Great Britain. A broad range of phenotypic and health-related information was collected from each participant, including physical measurements, lifestyle indicators, biomarkers in blood and urine, imaging, and routine health record data.
Recruitment	Participants of the UK Biobank aged from 40 to 69, who were registered with the NHS and living up to about 25 miles from one of the 22 study assessment centres were invited to participate in 2006-2010.
Ethics oversight	The UK Biobank has approval from the North West Multi-centre Research Ethics Committee (REC reference 13/NW/0157, https://www.ukbiobank.ac.uk/media/lcvbdoik/21-nw-0157-favourable-opinion-with-conditions-18-06-2021.pdf) as a Research Tissue Bank (RTB) approval and informed consent (https://www.ukbiobank.ac.uk/media/t22hbo35/consent-form.pdf) was provided by each participant. This approval means that researchers do not require separate ethical clearance and can operate under the RTB approval. This RTB approval was granted initially in 2011 and it is a renewal on a 5-yearly cycle; hence UK Biobank successfully applied to renew it in 2016 and 2021.<br><br>Use of the Icelandic data was approved by the National Bioethics Committee (VSN-15-169). All genotyped participants signed a written informed consent allowing the use of their samples and data in projects at deCODE genetics approved by the NBC. Data were anonymized and encrypted by a third-party system, approved and monitored by the Icelandic Data Protection Authority.

Note that full information on the approval of the study protocol must also be provided in the manuscript.

Field-specific reporting

Please select the one below that is the best fit for your research. If you are not sure, read the appropriate sections before making your selection.

☒ Life sciences ☐ Behavioural & social sciences ☐ Ecological, evolutionary & environmental sciences

For a reference copy of the document with all sections, see nature.com/documents/nr-reporting-summary-flat.pdf

Life sciences study design

All studies must disclose on these points even when the disclosure is negative.

Sample size	We used the full available sample with whole-exome sequencing and phenotype data in UK Biobank for discovery analyses.
Data exclusions	Only individuals failing standard genotyping quality control parameters, individuals of non-European ancestry or with missing phenotype or covariates were excluded from analysis. This decision was made prior to performing any downstream analysis.
Replication	We replicated our findings using independent data from an Icelandic cohort.
Randomization	The principle exposure in this study is naturally occurring genetic variants, meaning that we were unable to randomize the individuals in the study. To account for possible confounding, we used a linear mixed model and adjusted for technical and demographic covariates.
Blinding	Blinding is not applicable to this study, as it is an association study of rare genetic variation and not a randomized controlled trial. We did not deliver any intervention to the participants in this study.

Reporting for specific materials, systems and methods

We require information from authors about some types of materials, experimental systems and methods used in many studies. Here, indicate whether each material, system or method listed is relevant to your study. If you are not sure if a list item applies to your research, read the appropriate section before selecting a response.

Materials & experimental systems

n/a	Involved in the study
☒	☐ Antibodies
☒	☐ Eukaryotic cell lines
☒	☐ Palaeontology and archaeology
☒	☐ Animals and other organisms
☒	☐ Clinical data
☒	☐ Dual use research of concern
☒	☐ Plants

Methods

n/a	Involved in the study
☒	☐ ChIP-seq
☒	☐ Flow cytometry
☒	☐ MRI-based neuroimaging

Plants

Seed stocks	NA
Novel plant genotypes	NA
Authentication	NA
